# Fungal Contamination in Microalgal Cultivation: Biological and Biotechnological Aspects of Fungi-Microalgae Interaction

**DOI:** 10.3390/jof8101099

**Published:** 2022-10-18

**Authors:** Carmen Laezza, Giovanna Salbitani, Simona Carfagna

**Affiliations:** 1 Dipartimento di Agraria, Università di Napoli Federico II, Via Università 133, Portici, 80055 Napoli, Italy; 2 Dipartimento di Biologia, Università di Napoli Federico II, Via Cinthia 21, 80126 Napoli, Italy

**Keywords:** biological contaminants, fungi, microalgae, parasites

## Abstract

In the last few decades, the increasing interest in microalgae as sources of new biomolecules and environmental remediators stimulated scientists’ investigations and industrial applications. Nowadays, microalgae are exploited in different fields such as cosmeceuticals, nutraceuticals and as human and animal food supplements. Microalgae can be grown using various cultivation systems depending on their final application. One of the main problems in microalgae cultivations is the possible presence of biological contaminants. Fungi, among the main contaminants in microalgal cultures, are able to influence the production and quality of biomass significantly. Here, we describe fungal contamination considering both shortcomings and benefits of fungi-microalgae interactions, highlighting the biological aspects of this interaction and the possible biotechnological applications.

## 1. Introduction

Microalgae are photosynthetic unicellular or simple-multicellular microorganisms, smaller than 400 µm, that form the base of the entire aquatic food chain [1]. They have been able to adapt to a large range of temperatures, pH, salinities, and light intensities and this led them to evolve into a wide variety of species, colonizing many places on earth including extremophilic habitats [2,3,4]. In the last few decades, the increasing interest in these microorganisms stimulated scientists to investigate how to use them as sources of biomolecules or as sustainable solutions for environmental issues [5,6,7,8,9]. For instance, microalgae can be used for bioremediation [8,9,10], or as biofertilizers and biostimulants or biopesticides [7,11,12,13,14,15,16]. Due to their high concentrations of proteins, vitamins, and fatty acids, microalgae have been also evaluated as possible nutrients for different animals including both aquatic and terrestrial species [17,18,19,20,21,22,23,24]. Moreover, antioxidant molecules and pigments can have beneficial effects on animal and human wellness [25,26,27,28].

For biotechnological applications, very large volumes of microalgae are required to produce considerable amounts of biomass, which enhances the possibility of contaminations. The presence of biological contaminants can often cause a decrease in biomass production [29,30,31,32,33,34,35] and sometimes massive death of microalgae within cultures [36].

Thus, a balance in the coexistence of microalgae and contaminants should be pursued beneficially for large-scale cultivation. To this end, microalgae-mixed cultures and co-cultures are effective methods to use. In mixed cultures, microalgae are mixed with a varied spectrum of microorganisms [37,38,39,40,41,42]. Co-cultures consist of microalgae growing with another particular species that is usually effective to boost the production of algal biomass [20,43,44,45].

The interaction between microalgae and fungi is very interesting to consider even if less investigated than the interaction between microalgae and bacteria [46]. In commercial cultivations, fungi contamination is of particular concern [47]. On the other hand, according to recent literature, the microalgae-fungi interaction can be used to improve wastewater treatment, to facilitate cultivation management, and to produce valuable metabolites [20,48,49,50,51,52,53,54]. In fact, fungi are often used in co-cultures as they can promote the flocculation of microalgae, improving their harvesting without using more expensive chemical flocculants [37,55,56,57,58,59,60,61,62].

Here, we will review the mechanisms of interaction between fungi and microalgae in cultivation systems, highlighting some possible advantages for the industrial and biotechnological applications.

## 2. Overview on Biological Contaminants in Microalgal Cultivation Systems

Globally, more than 80% of algal biomass is generated in open reactors due to the low costs of these cultivation systems [62]. Nevertheless, the use of closed cultivation systems is expected to grow by 2024 because of their benefits [63]. Closed systems are mainly represented by photobioreactors (PBRs), in which temperature, light, pH, and nutrients are controlled, resulting in increased cell density and biomass collection [62]. However, both systems have their downsides. In open systems, light saturation and seasonal influences can affect algal biomass growth, making difficult the control of the culture. In closed systems, the cost of the equipment used to maintain constant cultivation parameters and sterilization is too expensive. Moreover, these two systems share the crucial challenge of contamination [64]. The interactions between microalgae and other organisms such as bacteria, fungi, viruses, and others are regularly present in nature [65]. Thus, it is common for these types of relationships also to develop in open systems and, to a smaller degree, in closed systems used for microalgae cultivation [66,67].

The groups of microorganisms considered as common contaminants in microalgae cultivation systems are grazers, bacteria, fungi, photosynthetic organisms, and viruses [46,68]. The relationships among these classes of organisms and microalgae can be: (i) mutualistic or symbiotic; (ii) commensalistic; (iii) parasitic. For instance, a symbiosis can be established between bacteria and microalgae (e.g., *Dunaliella salina* with *Marinobacter* sp., *Porphyridium purpureum* with *Halomonas* sp., *Chlamydomonas nivali* with *Mesorhizobiums* sp.), and they are often co-cultivated for many purposes [56,65]. Interesting results were achieved in terms of lipid production with the co-cultivation of *Characium* sp. And the heterotrophic bacterium, *Pseudomonas composti*, or with *Chlorella vulgaris* and *Stenotrophomona smaltophilia* [69,70]. Parasitism occurs when an organism steals a resource from another organism. For example, bacteria belonging to genera such as *Alteromonas*, *Flavobacterium*, *Bacillus*, *Pseudomonas* can attack microalgae by cell-to-cell contact or by releasing extracellular compounds [71,72,73], breaking the integrity of the microalgal cell walls, entering the cells, and destroying the DNA [74].

Another example of organisms competing with microalgae are grazers such as ciliates, rotifers, copepods, and Cladocera. These are larger than microalgae and can cause a rapid and significant depletion in microalgal biomass [36]. Other photosynthetic groups, such as competing microalgal species, can overwhelm the cultivated strain. Harmful microalgae are probably the most difficult form of contamination to control, since the biological and physical properties of the contaminant are very similar to those of the desired species [75].

Microalgae cultures may also be affected by several species of fungi [76]. *Chytridiomycetes* spp. are host-specific parasites and among the most pathogenic fungal groups for microalgal populations. The main concern in this case is that fungi can survive without their host if they have enough organic compounds to live on [77]. This explains why different fungal species are found in heterotrophic cultures in closed systems and why the possibility of a fungal outbreak in the cultivation may occur.

## 3. Fungi in Aquatic Environment

Fungal cells have a true nucleus, internal cell structures, and a cell wall. A unique property of their nuclear membrane and nucleolus is that they persist throughout the metaphase of cellular division, unlike animals and plants cells. An important structure of fungal cells is the wall that protects them from osmotic pressure and environmental stress and determines the cell shape. The fungal wall also prevents the intrusion of toxic macromolecules, and this may cause fungal resistance to certain fungicidal products [78]. Although the cell wall composition differs among the different fungal species, most varieties consist of: (1→3)-*β*-glucan, (1→6)-*β*-glucan, (1→3)-*α*-glucan, chitin, and glycoproteins [79].

A hypha is another fungi trait which consists of one or more cells surrounded by a cell wall. The multitude of cells forming the hypha is internally divided by cross-walls called ‘‘septa’’. This fungal structure is classified as a true hypha in molds (multicellular filamentous fungi) and pseudo-hyphae in yeasts (unicellular filamentous fungi). A mass of hyphae constitutes the thallus (vegetative body) of the fungus, composed of mycelium. These filaments branch out in all directions, thus colonizing wide spaces in their habitat.

In natural aquatic environments, the key function of fungi is the degradation of dead plants or other organic material. Decomposition is vital for the nutrient cycle, resulting in the production of fungal biomass, the formation of reproductive spores, and transformation products as dissolved organic matter [80]. This process enhances the palatability and nutritional quality of the litter for the invertebrates [81], consequently transferring energy and nutrients to higher trophic levels [82]. Nevertheless, the fungal role in the natural environment is still largely underexplored even if fungal presence has been frequently observed [6,83,84]. Different classes of fungi were identified also in artificial aquatic habitats such as: (i) urban wastewaters [85]; (ii) algal mass cultures [86]; (iii) hydroponic systems for plants [87].

**Figure 1:**
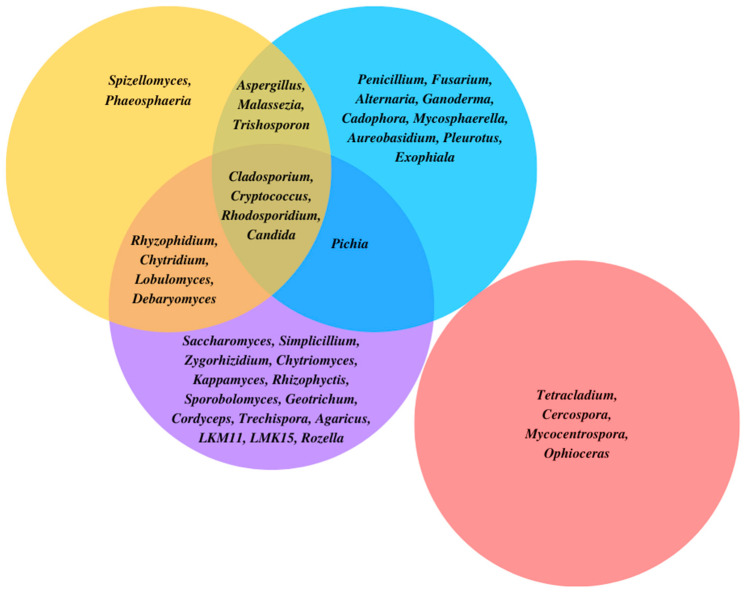
Predominant genera in different aquatic habitats. Yellow: coastal and oceanic environments; Blue: deep sea and sub-sea floor; Purple: lakes; Red: rivers, streams, and ponds.

Predominant genera of fungi in different aquatic habitats are shown in Figure 1. In these habitats, the fungi contribution and their interaction with other organisms are influenced by several abiotic factors such as nutrient availability, light, temperature, and pH. Visible light is an important source of energy for autotrophic organisms, and, although fungi are not photosynthetic organisms, they are affected by light. Over the last few years, some studies analyzed the effects of light on primary metabolic pathways, the production of secondary metabolites, and sporulation related to fungi [88]. Fungi exploit light to adapt to stressful conditions and to orient themselves in the environment and produce reproductive structures in the right place and the right time [88,89]. In addition, light has different effects on the biosynthesis of mycotoxin, depending on light intensity and wavelength, as well as on the species of fungi. In *Penicillium nordicum* and *Penicillium verrucosum*, blue (455–470 nm) and red (627 nm) wavelengths reduce the biosynthesis of the ochratoxin A and influence their growth and metabolism [90,91]. Other influencing factors for fungal growth are environmental temperature and pH [92]. In general, fungi prefer a liquid media with pH ranging from 3.0 to 8.0, with a growth optimum around pH 5.0 [92]. For instance, *Aspergillus* spp. are more tolerant to alkaline pH, while *Penicillium* spp. appear to be more tolerant to acidic pH [93]. Fungi can actively modify the pH of their environment, adapting it to their needs, by secreting acids or alkali [94]. The ability of pH change depends on the nutrient availability, the organic acids being produced, and the ability of the fungus to remove ammonium ions from media and to excrete H^+^ deriving from NH_4_^+^ assimilation [94,95]. Several pathogenic fungi acidify the environmental media as a strategy to damage host tissues [94]. Temperature is another environmental factor influencing metabolic functions of microorganisms [96]. Fungi can adapt at a relatively large range of temperatures; in aquatic environments (natural and not), they are found at temperatures ranging from 0 to 34 °C [97].

## 4. How Fungi Interact with Other Organisms in Aquatic Habitats

Although fungi are fundamental for aquatic food chains, they can also operate as parasites. Fungal parasitism can highly influence the dynamics that occur among the species that populate aquatic ecosystems [98]. Parasitism is not often clearly distinguishable from mutualism [99], but there is evidence that both parasitic and mutualistic fungal species exist. Fungi are considered among the most dangerous parasitic species for other organisms, as they can strongly reduce the number of parasitised individuals [77]. However, fungi are not obligate parasites and only use the host for completing their life cycle. The most common *modus operandi* of fungi when interfacing with other organisms is the release of metabolites. Fungi synthesize a broad spectrum of chemical compounds from either primary or secondary metabolism. Different secondary metabolites have been studied for their potential as pathogenic substances, such as mycotoxins, enzymes, siderophores, pigments, and others. Secondary metabolites released by species belonging to *Fusarium* and *Aspergillus* are able to activate specific gene clusters within bacteria, establishing either a mutualist or competitive interaction [100,101]. Antagonism between fungi and bacteria seems to be frequent, and it is related to nutrient competition. Nevertheless, synergistic interactions have also been demonstrated [87]. Baudy et al. [102] described the positive effect that bacteria can have on the competition occurring between different fungi species. Bacterial inhibition of fungal growth plays a pivotal role during fungi colonization, enabling fungal species with lower growth rates to colonize the aquatic environment under lower competitive pressure. Fungi also hold a relation with viruses that can be both mutualistic and antagonistic [87]. Recently, it was demonstrated that viruses can infect fungi in aquatic habitats [103,104]. However, further studies need to be conducted on this relation. Furthermore, both microalgae and fungi can be present in the same waterbody, and they are usually in competition for the nutritional resources [105]. In marine habitats, physical associations between fungi and microalgae have been reported to induce microalgal aggregation, while no data are available on their chemical interactions [56,84].

In summary, fungi can have a positive or negative impact on other organisms as they can be involved in a range of interactions shifting from cooperation to competition [106].

## 5. Fungi in Microalgae Cultures

Fungi can act as a competitor or symbiont with freshwater and marine microalgae cultures. There are many examples of spontaneous and induced fungal contamination of microalgae cultures (Table 1). Complete removal of undesired biological parasites at the industrial scale is neither cost-effective nor achievable [107].

Members of the Chytridiomycota are one of the most common fungal parasites associated with microalgae in both open and closed culture systems. Their hosts’ spectrum can be narrow or wide, depending on the species. This group of fungi causes huge losses in microalgae populations, and unfortunately is resistant to several disinfection techniques [77]. Aphelids are intracellular parasites that feed on microalgae and are close to the taxon of chytrids [126]. The group of Labyrinthulyds includes some important parasites of marine microalgae. However, they have not been described in commercial systems yet [77]. Zoosporic fungi negatively affect diatoms’ biomass production [127]. Many of these fungal pathogens caused the disappearance of several marine microalgae in specific areas of India, the USA, and Europe, and it is thought that these species can be even more harmful in commercial systems [128].

Fungal infection occurs in several crucial steps. The attack occurs within a few days, and fungi damage the microalgae’s cell walls. Specific enzymes are presumably involved in this stage. Subsequently, pathogens settle, encyst, and germinate inside the host. Microalgae usually lose their green color, turning brown. Young microalgae during their motile phase are not infected but are targeted as soon as they mature [129].

The nutrients present in the culture systems and that may facilitate the spread of fungal parasites are inorganic salts, potassium, magnesium, sulphate, phosphate. In hetero- and mixo-trophic microalgal cultures, the presence of organic substrates such as glucose or sucrose induces a significant growth in the fungal population and increases contamination. On the contrary, the presence of bicarbonate or galactose in the medium severely limits the contamination of microalgal cultures [108,130].

Generally, a molecular identification of fungal contamination is time-consuming and costly. Rather, strategies to eliminate or reduce contaminants should be applied. Several methods are used to deal with contamination. An example of a biological approach is the adoption of viruses attacking fungi as well as bacteria [131]. This is a viable strategy as long as viruses also parasitize microalgae culture. A most traditional, chemical resolution is the application of triticonazole and other commercial fungicides. However, these fungicides have been reported to be toxic also for some microalgae species [117].

In summary, it is of pivotal importance to manage the cleaning and sanitization phases correctly, to avoid large contaminations by pathogenic fungi, specifically in closed systems. Contamination can be caused by inadequate sterilization of facilities, control of inocula, culture media, water supply, and aeration gases [68]. Studying interactions and dynamics in fungi-microalgae relationships is becoming very important for optimizing yields and the productivity of algal factories.

## 6. Co-Culture of Fungi-Microalgae: The Biotechnological Use

The interaction between microalgae and fungi existed for more than 600 million years [132]. However, it was recently investigated since the fungi-microalgae consortium serves several biotechnological applications.

One of the most important advantages of co-cultures is related to microalgae harvesting. This can be economically challenging because of microalgae’s small size (2–40 μm), motility, negatively charged surface, and low cell density (0.3–0.5 g/L) [133,134]. In fact, harvesting can account for more than 50% of total production costs [52]. Different approaches have been used to collect microalgae, and each of them has evident limitations [52,54,135]. Fungi have potential as bio-flocculants since self-pelletization of fungi occurs in various fungal strains. This same property can lead to the increase in algal biomass collection [135].

**Figure 2:**
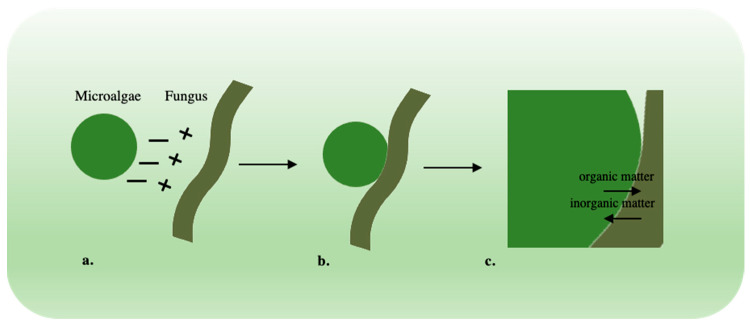
(**a**). Interaction between microalga (negative charge) and fungus (positive charge); (**b**). microalga entrapped among fungal hyphae; (**c**). nutrients exchange between microalga and fungus.

The co-pelletization is made possible since the surface of microalgae is negatively charged because of phosphoric, phosphodiester, amine, hydroxyl, and proton-active carboxylic functional groups, while the surface of fungi is positively charged by their surface containing polysaccharides [135]. This causes microalgae to be entrapped in fungal hyphae (Figure 2), ranging in diameter from 2 to 10 mm, where they aggregate, segregating from the surrounding liquid, which also facilitates microalgae recovery [52,56,136].

Fungal spores or fungal pellets can be included in the microalgae culture for a more efficient symbiosis and the consequent enhancement of co-pelletization. However, the technique is expensive as the pellet consumes nutrients. This technique would be more applicable when fungal growth is achieved without economic investment, as cultivating fungi or waste [137]. Costs of co-pelletization are further reduced as fungi consume sugars and nutrients produced by microalgae photosynthesis, and, in return, the algal biomass increases due to the retention of water and nutrients by fungi [52,138,139].

Different parameters can however affect the microalgae-fungi interaction in both positive and negative ways. Strain selection is one of the major factors to consider when developing an effective binary culture system. Assuming the primary partner is a well-defined microalgal species, the secondary partner (the fungus) needs to (i) be able to co-exist with this species; (ii) neither inhibit its growth nor be toxic for it; (iii) have an adequate communication (metabolite/peptide) profile; (iv) be able to use the primary species’ wastes as feedstock for its growth. In most cases, choosing two species already living in the same habitat can be a good starting point for the development of the fungi-microalgae consortium [52,139,140].

It is also important to consider when, during the cultivation, to add the fungal inoculum, and in which ratio with the primary partner to avoid overgrowth of either species at the expense of the other [51,141]. To limit growth imbalance, control of the amount of nutrient within the medium culture is also essential. It was previously established that the fungi-microalgae consortium usually requires mixotrophic conditions as the fungi are obligate heterotrophs, and green microalgae are photoautotrophs [142,143]. This requires fungal growth on expensive nutrients such as glucose. However, cheaper compounds such as sucrose, glycerol, sodium acetate can be used as alternatives to glucose [45], and fungal growth on food wastes is being now attempted, as anticipated. In all cases, it is fundamental that microalgae and fungi do not reach the peak of their growth during the same experimental phase [144] to avoid the overwhelming of one or the other species.

The co-cultivation of microalgae and fungi is also strongly dependent on factors such as pH, temperature, agitation rate, etc. A more acidic pH favors an enhanced growth of fungal hyphae [145]. However, the acidic pH of the growth medium frequently becomes more basic as bicarbonate (HCO^−^_3_) is commonly converted to CO_2_ and hydroxide ions (OH^−^) [146]. Microalgae consume CO_2_, inducing an excess of OH^−^ within the growth medium, resulting in a pH increases [147].

Alterations in temperature can also make the system unstable, altering the microalgae lipid profile. Co-culturing fungi and microalgae that are similarly tolerant to stresses (e.g., halotolerant or thermophile) is often suggested [2,4,148].

The fungi-microalgae binary system has been adopted for several scopes. Since wastewaters pose a great risk to human health and ecosystems, as they carry many toxic substances, the fungi-microalgae co-culture was recently considered as a viable approach for their treatments and remediation [149]. In water, as previously mentioned, inorganic carbon is mainly present in the form of HCO^−^_3_ that microalgae can actively absorb and convert directly into CO_2_ [146]. Subsequently, they convert CO_2_ into organic compounds, via photosynthesis, further releasing oxygen. Oxygen will be used by fungi for their respiration process and the organic compounds for their growth. 

In most cases, wastewater needs to be purified from heavy metals. It was previously demonstrated that microalgae can face the presence of high concentrations of heavy metals when grown with fungi, as fungi secrete organic acids that create favorable acidic conditions for the transformation of these toxic molecules into less toxic compounds [150]. Fungi and microalgae walls contain functional substances such as cellulose, proteins, and other polymers, and they may help by absorbing other elements due to mechanisms such as electrostatic interactions, ion exchange, and chelation/complexation [45]. Furthermore, fungi are also able to release extracellular polymeric substances (EPS) that can bind metal ions, playing an important role in protecting the microalgae from stress and preventing a reduction in total biomass in stressful conditions [151]. Wang et al. [52] demonstrated that the synergic action between *Synechocystis* sp. PCC6803 and *A. fumigatus* induces 98% adsorption and immobilization of Cd(II) within wastewater.

Fungi-microalgae systems can be also involved in other wastewater treatments which involve the purification from antibiotics, food organics, and nitrogen (N) and phosphorus (P) [48,152]. Microalgae are not able directly to utilize some substrates, and a binary culture with fungi might represent a suitable option to overcome this obstacle. For example, one-step co-cultivation of *C. pyrenoidosa* with *A. oryzae* exhibited an optimal removal efficiency of the chemical oxygen demand (COD), total nitrogen (TN) and total phosphorus (TP) [153].

The microalgae-fungi consortium made by *Chlorella vulgaris* and *P. geesteranus* prompted a high-rate removal of COD, TN, and TP, as well. Moreover, the presence of the filamentous fungus enhanced growth performance and photosynthesis of *C. vulgaris* resulting in a consistent CO_2_ removal ability [54]. Another culture binary system demonstrating effectiveness is *Chrorella vulgaris* and *Aspergillus oryzae*. The fungi-microalgae pellet originating by this symbiosis presented a remarkable adsorption capacity of sulfamethazine (SMZ), sulfamonomethoxine (SMM), and sulfamethoxazole (SMX) [20].

Cooperation between microalgae and fungi is not only convenient for wastewater treatments, but also for producing a wide range of distinctive substances of use in food, cosmetic, and renewable energy industries. For instance, the demand for fish oil, rich in polyunsaturated omega-3 fatty acids (PUFA), is constantly growing, while over-fishing is becoming an urgent issue to cope with. Marine microalgae and diatoms produce an impressive amount of PUFA, among which the most important are Eicosapentaenoic acid (EPA) and Docosahexaenoic acid (DHA) [154]. However, up to now, no oil-producing microalgae possess a good ratio of these two compounds. Co-culturing microalgae with fungi has been recognized as a favorable strategy to solve this problem alternatively. For example, the culture system composed by *P. tricornutum* and *A. limacinum* produced EPA and DHA in advantageous ratios [155]. Carotenoids’ production is also enhanced when co-culturing fungi and microalgae. Carotenoids produced by co-cultivation of *C. vulgaris* and *R. glutinis* were higher than those made by each culture [156]. 

Microalgae are considered one of the best options for generating energy without impacting the environment, and they gained attention as suitable alternative bio-fuel sources. Moreover, in this case, co-cultivation with fungi may further increase the value of microalgae as energy crops. For example, co-cultures of *C. vulgaris* and *P. geesteranus* [54] and of various microalgae with *A. niger* and *T. reesei* [157] were considered valuable sources of bioenergy. Indeed, when microalgae were grown with the two filamentous fungi, they exhibited increased cellulase activity. This improves the hydrolysis process of cellulosic materials which is considered a key point for bioethanol production [157].

## 7. Concluding Remarks

The fungi-microalgae relationship needs to be better explored in systems of microalgae cultivation as some fungal species can contaminate algal culture, leading to an extensive reduction in the algal population. On the other hand, the relationship between fungi and microalgae is a mechanism that could potentially be used for different industrial purposes to develop productions cheaper and more sustainable than with other methods. 

## Figures and Tables

**Table 1:** Parasites reported for microalgae in laboratory culture and open raceways.

System	Microalgal Host	Parasite’s Species	References
Laboratory culture	*Haematococcus pluvialis*	*Paraphysoderma sedebokerensis* (Chytrid)	[108,109,110]
	*Chlorella vulgaris*	*Aspergillus niger*	[69]
	*Chlorella vulgaris*	*Mucor* sp.	[111]
	Various diatoms	*Chytriomyces* sp. and *Zygorhizidium* sp. (Chytrid)	[112]
	*Scenedesmus* sp.	*Amoeboaphelidium protococcarum* (Aphelid)	[113]
	*Scenedesmus* sp.	*Phlyctidium scenedesmi*	[114]
	*Asterionella Formosa*	*Rhizophydium planktonicum* (Chytrid)	[115]
	*Nannochloropsis oceanica*	*Mortierella elongata*	[116]
	*Nannochloropsis oceanica*	*Aspergillus sydowii*	[117]
	*Chlorococcorum minutum*	*Rhizophydium algavorum* (Chytrid)	[118]
	*Spirulina platensis*	*Rhodotorula glutinis*	[119]
	*Closterium* sp.	*Leptophyrs vorax* (Amoebae/Endomyxa)	[120]
Mass culture	*Scenedesmus* sp.	*Phlyctidium scenedesmi* (Chytrid)	[114,121]
	*Scenedesmus* sp.	*Amoeboaphelidium protococcarum* (Aphelid)	[122]
	*Scenedesmus* sp.	*Rhizophidium* sp.	[123]
	*Scenedesmus dimorphus*	*Amoeboaphelidium protococcarum*	[124]
	*Grasiella* sp.	*Amoeboaphelidium protococcarum* (Aphelid) and *Rhizophydium scenedesmi* (Chytrid)	[125]
